# Strain-Dependent Consequences of Zika Virus Infection and Differential Impact on Neural Development

**DOI:** 10.3390/v10100550

**Published:** 2018-10-09

**Authors:** Forrest T. Goodfellow, Katherine A. Willard, Xian Wu, Shelley Scoville, Steven L. Stice, Melinda A. Brindley

**Affiliations:** 1 Department of Animal and Dairy Science, Regenerative Bioscience Center, College of Agriculture and Environmental Science, University of Georgia, Athens, GA 30602, USA; forrestgoodfellow@gmail.com (F.T.G.); xian.wu@nih.gov (X.W.); 2 Department of Infectious Diseases, College of Veterinary Medicine, University of Georgia, Athens, GA 30602, USA; katherine.willard@duke.edu; 3 ArunA Biomedical, Athens, GA 30602, USA; sscoville@arunabio.com; 4 Department of Infectious Diseases, Department of Population Health, Center for Vaccines and Immunology, College of Veterinary Medicine, University of Georgia, Athens, GA 30602, USA

**Keywords:** Zika virus, neural progenitor cells, neurons

## Abstract

Maternal infection with Zika virus (ZIKV) during pregnancy can result in neonatal abnormalities, including neurological dysfunction and microcephaly. Experimental models of congenital Zika syndrome identified neural progenitor cells as a target of viral infection. Neural progenitor cells are responsible for populating the developing central nervous system with neurons and glia. Neural progenitor dysfunction can lead to severe birth defects, namely, lissencephaly, microcephaly, and cognitive deficits. For this study, the consequences of ZIKV infection in human pluripotent stem cell-derived neural progenitor (hNP) cells and neurons were evaluated. ZIKV isolates from Asian and African lineages displayed lineage-specific replication kinetics, cytopathic effects, and impacts on hNP function and neuronal differentiation. The currently circulating ZIKV isolates exhibit a unique profile of virulence, cytopathic effect, and impaired cellular functions that likely contribute to the pathological mechanism of congenital Zika syndrome. The authors found that infection with Asian-lineage ZIKV isolates impaired the proliferation and migration of hNP cells, and neuron maturation. In contrast, the African-lineage infections resulted in abrupt and extensive cell death. This work furthers the understanding of ZIKV-induced brain pathology.

## 1. Introduction

Zika virus (ZIKV) was recognized in the 1950s as an arbovirus that could infect humans, though it garnered little attention until the substantial outbreaks in French Polynesia and other South Pacific islands were reported in 2013–2014 [1,2]. ZIKV causes mild febrile illness, joint pain, and conjunctivitis when symptomatic, yet remains asymptomatic in 80% of infected individuals [3]. Beginning in 2016, an epidemic of ZIKV in Brazil established an evidentiary link between congenital infection and placental insufficiency, spontaneous abortion, ocular defects, and microcephaly [4,5,6,7]. However, these severe clinical manifestations of congenital ZIKV infection had not been previously reported in areas where ZIKV was endemic. A phylogenetic comparison of ZIKV isolates demonstrated a distinction between older African-lineage isolates of the virus and contemporary Asian-lineage isolates [8]. Further characterization of ZIKV isolates that are directly associated with congenital abnormalities suggests that genetic mutations distinguishing African-lineage and Asian-lineage ZIKV isolates may be responsible for the unique pathologies observed in Latin America.

Efforts to understand the pathological mechanism of congenital ZIKV infection were initiated using in vitro and in vivo model systems. Murine models of vector-borne, vertical, and sexual transmission were established [9,10,11,12,13]. Both vertically transmitted infections and direct infection of the developing mouse brain highlighted the susceptibility of human neural progenitor (hNP) cells and immature neurons to ZIKV infection [13,14,15]. This neurotropism was confirmed using in vitro organoid and 2D-cultures of hNP cells derived from pluripotent stem cells [16,17,18,19,20,21]. Experimental models of congenital ZIKV syndrome prompted the current hypothesis that ZIKV infects and damages the developing central nervous system (CNS) and, to a lesser extent, the peripheral nervous system (PNS) [20,22,23,24]. Initial studies suggest that African-lineage ZIKV isolates cause more profound cell death in hNP cells and neurons than Asian-lineage ZIKV [25].

To further address the lineage-specific effects of ZIKV infection, the authors employed a representative in vitro model of CNS development to directly evaluate the effects of ZIKV infection using both African-lineage and Asian-lineage isolates of ZIKV. Previously, the authors demonstrated that hNP cells could yield mature neurons in a predictable and well-defined manner representative of in vivo human development [26,27,28]. In vitro culture of hNP cells and hNP-derived neurons enabled the evaluation of viral replication and cytopathic effect of both Asian and African lineages of ZIKV. Although African isolates displayed more rapid viral replication and detriment to cell viability, the Asian isolates disrupted key functional aspects of hNP cells and hNP-derived neurons including proliferation, migration, and neurite outgrowth. The authors surmised that the less dramatic cell death allowed for a population of functionally damaged neurons to remain. Deficient neural maturation caused by the Asian lineages correlates to pronounced effects on in vivo human neural development. The defects in hNP maturation due to Asian-lineage infections increase the understanding of the underlying mechanisms of CNS malformation resulting from congenital ZIKV infection.

## 2. Materials and Methods

### 2.1. Culture and In Vitro Differentiation of hNP Cells

hPSC line WA09 (WiCell 0062, Madison, WI, USA) was used to derive hNP cells (hNP1^TM^ 00001) as previously described and was obtained from ArunA Biomedical, Inc. (Athens, GA, USA) [29]. hNP cells were thawed in proliferation medium containing AB2^TM^ basal medium supplemented with ANS^TM^ neural supplement (both from ArunA Biomedical Inc.), 2 mM l-glutamine (Gibco, Waltham, MA, USA), 2 U/mL penicillin (Gibco), 2 μg/mL streptomycin (Gibco), 20 ng/mL fibroblast growth factor 2 (FGF2) (R&D Systems Inc. Inc., Minneapolis, MN, USA), and 10 ng/mL leukemia inhibitory factor (LIF) (Millipore, Billerica, MA, USA) and subsequently plated on cell culture dishes coated with Matrigel 1:100 (B&D Biosciences, Bedford, MA, USA). Differentiation of hNP cultures was induced by substituting the proliferation medium with differentiation medium (the proliferation medium lacking FGF2). Cultures were maintained for up to 28 days in vitro (DIV) with the fresh differentiation medium applied every two or three days. All cell cultures were maintained at 37 °C in a humidified incubator with a 5% CO_2_ atmosphere.

### 2.2. ZIKV Stock Production

ZIKV stocks were grown in Vero cells. Cell supernatant was collected when cells showed >80% cytopathic effect. Supernatants were cleared of cell debris (5000× *g*, 5 min, 4 °C), aliquoted into cryovials, and frozen at −80 °C for one week before titers were determined. The amount of infectious virus was quantified by either TCID_50_ or plaque assays. African isolate MR766 was derived from the original ZIKV isolation and underwent extensive passaging in both mice and tissue culture cells. African isolate IbH 30656 (VR-1829™, ATCC) was also extensively passaged in cell culture before it was obtained. The IbH stock was obtained from ATCC. Asian isolate MEX1-44 was isolated from a mosquito in Chiapas, Mexico, and the virus was obtained after being passaged four times. The lab passaged the virus three additional times in Vero cells before completing these studies. Asian isolate SPH was isolated from a male ZIKV patient in Brazil. The virus was passaged two times in Vero cells before the stock was received, and the virus was passaged three times in Vero cells before performing the experiments. Prior to experimentation, all ZIKV isolates tested negative for Mycoplasma contamination (MycoSensor PCR Assay Kit, Agilent, West Cedar Creek, TX, USA).

### 2.3. ZIKV Infection of hNP Cells and Neurons

hNP cells, 14 DIV nascent neurons, or 28 DIV mature neurons were seeded into 12-well tissue culture treated dishes for viral replication assays at a density of 400,000 cells/well or Costar^®^ 96-well cell culture plates for viability and proliferation assays at a density of 30,000 cells/well. In all cases, wells were treated with Matrigel 1:200 (B&D) for one hour and rinsed with phosphate buffered saline (PBS) prior to plating. Cells adhered to the plate prior to ZIKV infection (12 h). Cells were infected by multiplicity of infection (MOI) ranging from 0.1 to 10. Viral inoculum was removed 12 h following infection. The cells were washed once with PBS and restored with the respective culture medium.

### 2.4. Virus Quantification

ZIKV isolates MR766, IbH, and MEX1-44 produced easily discernable plaques on Vero cells and therefore titers were determined using plaque assays. The differential plaque morphologies may be a result of the variation of amino acid deletions in the E protein glycosylation sites, which have been previously noted [30]. For plaque assays, Vero cells were infected with 10-fold serial dilutions of sample for 1–2 h. The inoculum was then removed, replaced with 1.5% semi-solid agar overlay, and the cells were incubated for 4–5 days at 37 °C, 5% CO_2_. The cells were fixed in 4% formalin in PBS and stained with crystal violet, and the number of viral plaques was enumerated. ZIKV-SPH stocks titers were determined by 50% tissue culture infectious dose (TCID_50_) titration on Vero cells according to the Spearmann–Karber method and scored 6–7 days later.

### 2.5. Cell Viability Assay

Cell viability was measured either two or six days following infection with CellTiter-Glo^®^ Luminescent Cell Viability Assay (Promega, Fitchburg, WA, USA) according to manufacturer specifications. Briefly, cells were lysed in CellTiter-Glo^®^ Reagent. After a 15-min incubation, luminescence was measured with a GloMax-96 Microplate Luminometer (Promega).

### 2.6. Immunocytochemistry to Assess Viral Infection or Proliferation

hNP, neurons, or Vero cells were fixed with 4% paraformaldehyde 48 h following infection by applying 100 μL of warm (37 °C) 8% paraformaldehyde solution to wells containing 100 μL of medium and incubated at room temperature for 20 min [31]. Following fixation, cells were washed three times with PBS. The following steps were performed with the epMotion^®^ 5073l liquid handling station (Eppendorf, Hauppauge, NY, USA). Cells were permeabilized with 0.5% saponin in PBS and blocked for 1 h in 0.1% saponin in PBS containing 2% bovine serum albumin (BSA). Anti-Ki67 antibody (ab15580) (ABCAM, Cambridge, MA, USA) or mouse anti-flavivirus group antigen monoclonal antibody (MAB10216) (Millipore, Temecula, CA, USA) were diluted 1:1000 or 1:400 respectively, in 0.1% saponin containing 2% BSA and incubated with cells for 2 h. Following incubation with these primary antibodies, cells were washed three times with 0.1% saponin containing 2% BSA and incubated with a 1:1000 dilution DyLight^®^ 488-conjugated donkey anti-rabbit IgG secondary antibody in 0.1% saponin containing 2% BSA for 1 h at room temperature while protected from light. Cells were then incubated in 0.1% Hoechst 33342 dye in 0.1% saponin containing 2% BSA for 20 min, then washed three times with PBS, and stored at 4 °C. Images and quantifications were done with the Thermo Scientific ArrayScanVTI HCS Reader (Thermo Fisher Scientific/Cellomics, Waltham, MA, USA). A minimum of eight wells per treatment group and three biological replicates were analyzed.

### 2.7. ORIS^TM^ Cell Migration Assay

60,000 hNP cells were seeded onto Oris Cell Migration Assay plates with seeding stoppers. After 12 h, wells were infected with ZIKV at an MOI of 10, 1, or 0.1. At 12 h post-infection, the seeding stoppers were removed, cells were washed once with PBS, and fresh differentiation medium was applied to all wells. Cells were allowed to migrate into the exclusion zone for 48 h and were then fixed and stained following the method previously stated to assess cellular proliferation. The migration assay was quantified with the attachment of the Oris^TM^ detection mask and the Thermo Scientific ArrayScanVTI HCS Reader (Thermo Fisher Scientific/Cellomics).

### 2.8. Neurite Outgrowth Assay

The neurite outgrowth assay was conducted as previously described [32]. Briefly, 28 DIV neurons were seeded onto Costar^®^ 96-well cell culture plates at a density of 15,000 cells per well. Cells were infected with ZIKV 12 h after plating, then the culture medium was washed and refreshed after an additional 12 h. At 48 h following infection, cells were fixed and stained with Anti-MAP2 antibody (5622) (Millipore, Temecula, CA, USA) at a dilution of 1:200. Following incubation in primary antibodies, DyLight^®^ 488-conjugated donkey anti-rabbit IgG secondary antibody and 0.1% Hoechst 33,342 dye were used following an identical method as the one used for the hNP immunocytochemistry assays reported in this study.

### 2.9. Statistics

All analyses were conducted in *R* [33]. All measurements were compared by ANOVA and Tukey honest significant difference (HSD) test. A *p*-value of <0.05 indicated statistically significant differences between groups.

## 3. Results

### 3.1. Isolate-Specific ZIKV Growth and Cytotoxicity in Human Neural Progenitor Cells

**Figure 1:**
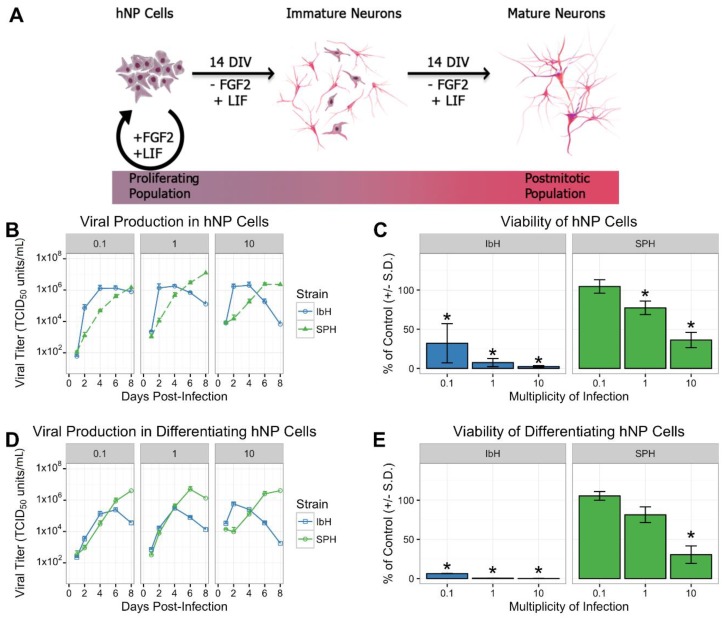
Zika virus (ZIKV) isolate-specific growth and cytotoxicity in human pluripotent stem cell-derived neural progenitor (hNP) cells at six days post-infection. (**A**) hNP cells are maintained as a proliferating population in media with fibroblast growth factor (FGF) and leukemia inhibitory factor (LIF). Withdrawal of FGF and LIF from the media leads to differentiation and a population of immature neurons after 14 DIV, then post-mitotic neurons exclusively emerge after 28 DIV. (**B**–**E**) African-lineage ZIKV isolate (IbH) grew robustly and induced cell death in undifferentiated hNP cells and differentiating hNP cells, while Asian isolate (SPH) replicated more slowly with less extensive cell death. * demonstrates *p* < 0.05.

Proliferating naïve hNP and differentiating hNP cells were infected with multiple isolates of ZIKV. As previously demonstrated, hNP cells self-renew when the culture medium contains FGF2 and LIF (Figure 1A) and neuronal differentiation is induced by the removal of FGF2 [34]. Two prototypical isolates of African-lineage ZIKV, MR766 and IbH [35], were juxtaposed against two contemporary ZIKV isolates of the Asian lineage, MEX1-44 and SPH [30]. Infection with African and Asian isolates resulted in differential temporal dynamics and peak titers. African isolates (MR766 and IbH) produced high viral titers within four days of initial infection and subsequently diminished (Figure 1B and Figure S1A). Asian isolates (MEX1-44 and SPH) demonstrated delayed growth, but ultimately achieved equal or greater peak titers compared to African isolates (Figure 1B and Figure S1A). In addition to robust virion production, both African and Asian isolates significantly reduced hNP and differentiating hNP viability six days post-infection compared to uninfected control cells (Figure 1C,E and Figure S1B,D). African stains were significantly more cytopathic than Asian isolates at comparable MOIs (Supplementary Tables S1 and S2). This result complemented the observed decrease in viral titers over time in the African-lineage infections. Cumulatively, ZIKV infection has an isolate-specific impact on viral replication and viability in both hNP and differentiating hNP cells.

### 3.2. Isolate-Specific Cell Death and Growth in hNP-Derived Neurons

**Figure 2:**
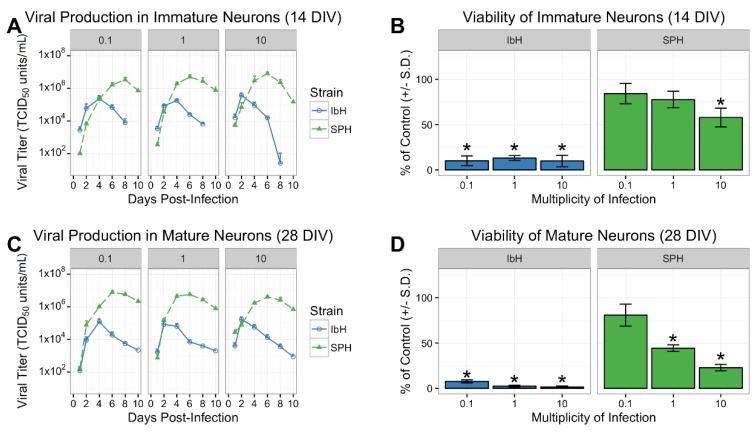
ZIKV isolate-specific growth and cytotoxicity in human neurons. (**A,B**) Viral replication and viability of hNP-derived nascent neurons (14 DIV) and (**C,D**) mature neurons (28 DIV) six days post-infection. * demonstrates *p* < 0.05.

The differentiation process from hNP to highly enriched mature neurons requires 28 days (28 DIV), whereas nascent neurons are evident halfway through the process (14 DIV). The authors, using their well-characterized hNP to neuron differentiation process, could characterize the pathogenic effects of ZIKV infection on populations of both immature and mature neurons. After 14 DIV in the absence of FGF2, hNP cell cultures presented a neuronal phenotype with reduced SOX1 expression and a portion of HuC/HuD+ and βIII-tubulin+ cells, indicative of maturing neurons (Figure 1A) [28]. The full 28 DIV of differentiation in vitro resulted in a highly homogeneous population of post-mitotic and mature neurons characterized by microtubule-associated protein 2 (MAP2) expression (Figure 1A) [34,36]. One African Zika isolate, IbH, and one Asian isolate, SPH, were selected to evaluate the isolate-specific effects of ZIKV on 14 DIV and 28 DIV neurons. The phenotypic differences between ZIKV lineages were again apparent when immature neurons were infected with African and Asian ZIKV. The African isolate, IbH, quickly reached peak viral production four days following infection. Peak IbH titers were followed by a marked reduction in viral production caused by virus-induced death of the cell population (Figure 2A,B). In contrast, cells infected with SPH continued to produce virions, resulting in higher viral titers peaking six or eight days post-infection. SPH-induced cell death was less apparent than with IbH, resulting in more viable immature neurons six days post-infection (Figure 2A,B). Mature neurons infected with SPH produced similar phenotypes to the nascent 14 DIV neurons (Figure 2C,D). Altogether, both isolates of the ZIKV effectively replicated in immature and mature neurons, although the Asian lineage produced higher viral titers while inducing less cell death.

### 3.3. ZIKV Infects Neural Progenitor Cells and Mature Neurons

**Figure 3:**
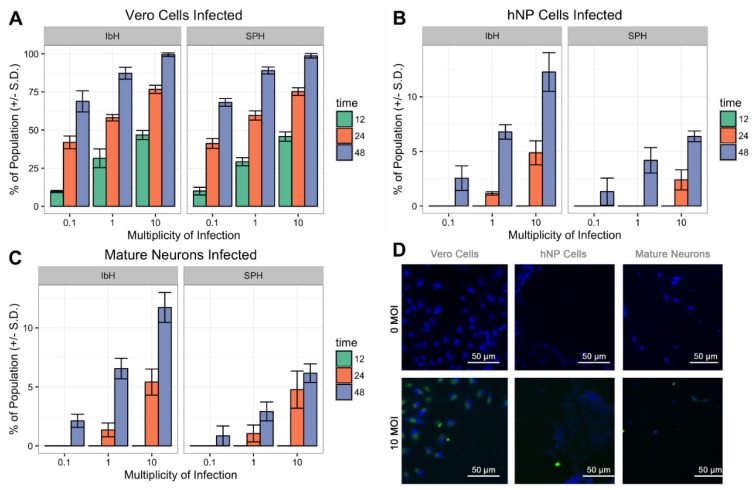
Isolate-dependent ability of ZIKV to infect hNP cells and mature neurons. (**A**) ZIKV isolates IbH and SPH readily infect Vero cells within 48 h. (**B**) hNP cells are more susceptible to IbH infection than SPH infection, however neither isolate infected more than 13% of the population. (**C**) Mature neurons (28 DIV) were similarly more susceptible to ZIKV IbH infection than SPH infection. (**D**) Non-infected (0 MOI) did not demonstrate ZIKV E protein’s presence, but infected (SPH 10 MOI) cells after 48 h did contain ZIKV E protein in all three cell types.

The differences observed in cell viability between the ZIKV lineages may be influenced by the isolates’ abilities to initially infect the cells. A previous study found that African isolates were able to infect significantly more hNP cells than an Asian isolate [37,38]. To evaluate the ability of two prototypical Asian and African ZIKV isolates to infect hNP cells and mature neurons, the number of hNP cell or neurons containing ZIKV E protein was compared to infected Vero cells 12 h after infection. Vero cells readily produced viral proteins, and pervasive infection was observed independent of the ZIKV isolate (Figure 3A,D). hNP cells and 28 DIV mature neurons displayed lower susceptibility/permissivity relative to Vero cells. Only a fraction of the hNP cells and 28 DIV neurons expressed ZIKV E protein, and lineage-specific susceptibility was observed in these cell lines. The high MOI infection (MOI 10) of hNP cells resulted in 13% of the IbH-infected cells expressing ZIKV E protein after 48 h, whereas only 7% of hNPs and 28 DIV neurons were infected by Asian isolate SPH (Figure 3B–D).

### 3.4. Neural Progenitor Cell Proliferation and Migration Decreases after ZIKV Infection

**Figure 4:**
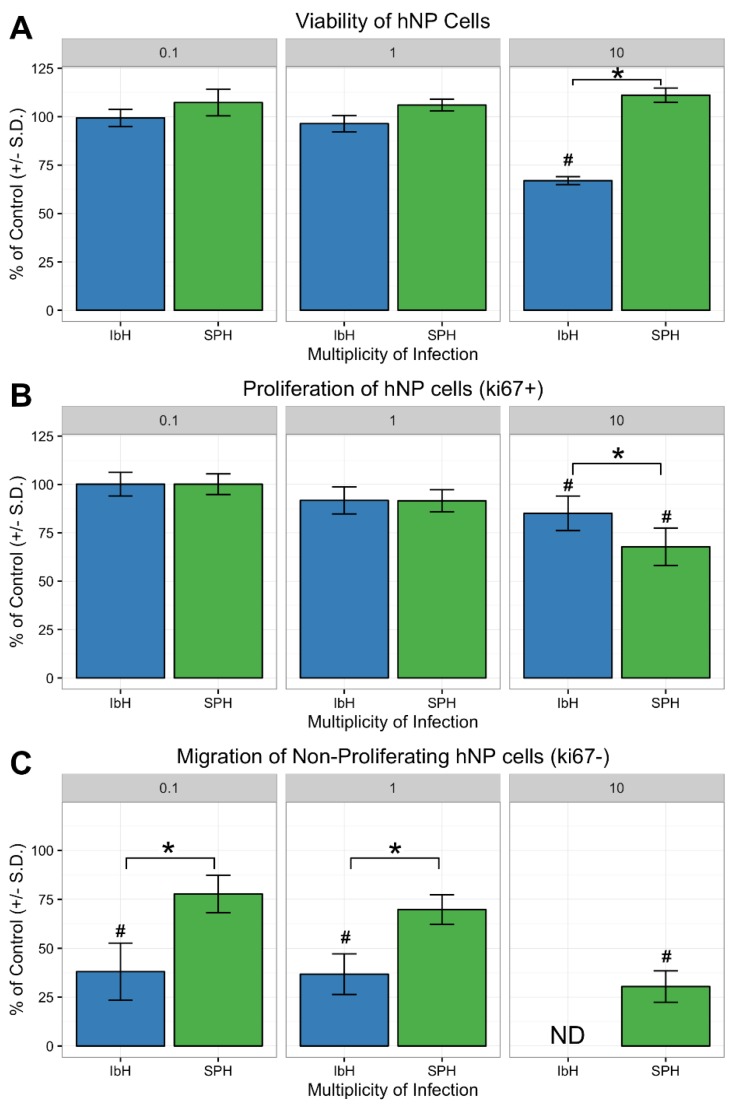
ZIKV infection decreased hNP cell proliferation and migration. (**A**) Only infection with MOI 10 of African-lineage ZIKV demonstrated a significant impact on cell viability after 48 h, whereas infection with SPH did not decrease viability. (**B**) The proliferation of hNP cells after ZIKV infection was significantly decreased following infection with SPH as determined by quantification of Ki67 expression. (**C**) The migration of hNP cells was disrupted by both IbH and SPH infections. IbH infection severely inhibited migration at MOI 10 to such an extent that no acceptable data were collected (shown as ND). * demonstrates *p* < 0.05 significance between IbH and SPH and # demonstrates *p* < 0.05 significance difference from non-infected control.

Proper hNP cell proliferation and migration are two fundamental cellular activities needed to construct the human cerebral cortex [26]. The impact of ZIKV infection on hNP cell function was evaluated 48 h after infection. At this time point, hNP cells infected with both IbH and SPH exhibited significant viral replication, yet minimal impact on cell viability (Figure 1B and Figure 4A). Proliferation of hNP cells diminished when infected with African and Asian ZIKV isolates, indicated by a decrease in the number of Ki67 positive cells (Figure 4B). However, infection with SPH decreased hNP cell proliferation without impacting cell viability (Figure 4A,B). hNP cell motility was evaluated by enumerating the cells migrating into the exclusion zone. hNP cells infected with either IbH or SPH exhibited a reduced ability to migrate compared to non-infected hNP cells (Figure 4C). IbH-infected populations had significantly fewer mobile hNP cells at all MOIs. In contrast, SPH-infected hNP cells only significantly impaired migration when infected at a high MOI (Figure 4C). Overall, ZIKV infection impacted the proliferation and migration of hNP cells, and these impacts occurred prior to reduced cell viability resulting from ZIKV infection.

### 3.5. Neuronal Maturation is Hindered by ZIKV Infection

**Figure 5:**
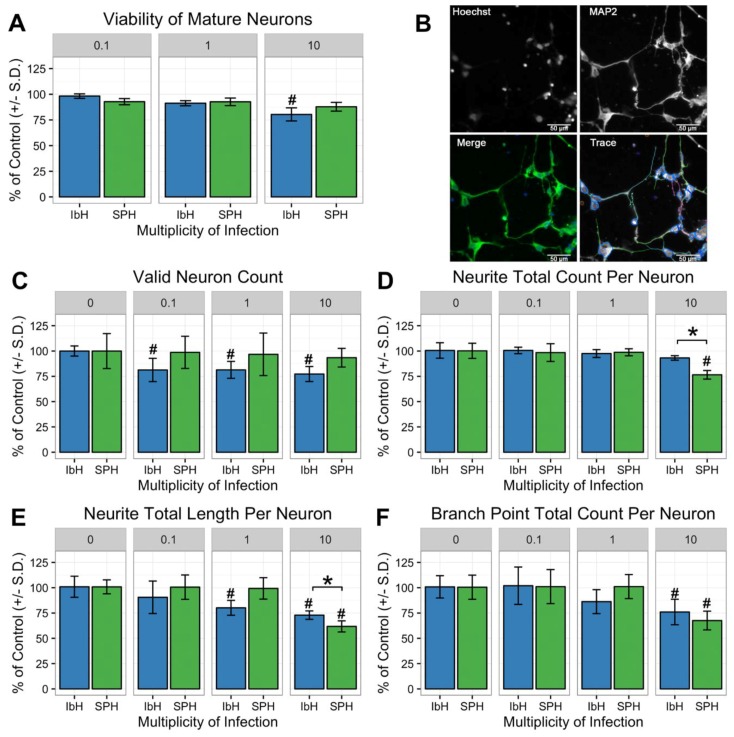
ZIKV infection perturbs neurite outgrowth in human neural progenitor cell-derived neurons. (**A**) At 48 h post-infection, both African (IbH) and Asian (SPH) isolates had minimal impact on neuron viability. (**B**) Representative images of neurite outgrowth quantification. Hoechst stain labels nuclei of neurons and MAP2 identifies neurites. Both images contribute to the enumeration and quantification of neurite outgrowth characteristics. Scale bar = 50 micrometers (**C**–**F**). Infection with Asian-lineage ZIKV (SPH) did not reduce the number of valid neurons observed, yet infection with SPH (MOI 10) proved detrimental to neurite outgrowth by decreasing the quantity, length, and number of branch points per neuron. * demonstrates *p* < 0.05 significance between IbH and SPH and # demonstrates *p* < 0.05 significance difference from non-infected control.

Neurite outgrowth of cortical neurons is a critical step near the conclusion of neural maturation, and pathological perturbation of neurite outgrowth contributes to defects in cognitive function [39]. The impact of ZIKV infection on neurite outgrowth was assessed prior to significant viral-induced cell death. Both IbH and SPH Zika isolates actively produced virus in mature 28 DIV neurons 48 h after initial infection despite a relatively small proportion of the cell population exhibiting an active infection (Figure 3C and Figure 2C). The acute infection with IbH, but not SPH, induced significant cell death at high MOI (Figure 5A,C). Infection of 28 DIV neurons with SPH significantly diminished the number of neurites, attenuated the neurite length, and decreased the number of branch points (Figure 5B–F). The total number of neurites extending from neurons infected with IbH was unaltered, but the length and number of branch points were decreased. Infection with Asian isolate SPH did not impact cell viability but altered the neurite outgrowth. Collectively, Asian isolate ZIKV infection impacted neurite outgrowth prior to decreased viability of the infected 28 DIV neurons. This result suggested an exceptional ability of the Asian isolates to functionally damage populations of neurons differently than African isolates.

## 4. Discussion

hNP cells and hNP-derived neurons are advantageous when assessing developmental malformations and provide a platform to examine the effects toxins and pathogens exert in a controlled, homogenous, and reproducible setting [39]. In vitro assays designed to evaluate critical functions of hNP and hNP-derived neurons provide critical insight into how environmental stresses and pathogens can alter normal functions including cell migration and neurite outgrowth [28,39,40]. In order to evaluate the impact of Asian and African isolates of ZIKV on human neural cell development, both undifferentiated hNP cells and hNP-derived neurons were infected to observe ZIKV-induced changes in differentiation, proliferation, migration, and neurite outgrowth.

Initially, the authors compared African and Asian isolates’ abilities to infect, replicate, and induce cell death in hNP cells and neurons. Genetic divergence between African and Asian isolates of ZIKV has been implicated in differential infection phenotypes [8,35,38]. Embryonic mice infected with African ZIKV isolates exhibited more pronounced apoptosis and a larger decrease in progenitor cell proliferation than mice infected with an Asian isolate [25]. African isolates of ZIKV induced higher chicken embryo mortality than Asian isolates [30]. These observations were confirmed with in vitro model systems using hNP cells and other cell types within the developing brain derived from pluripotent stem cells. However, the possibility remains that pluripotent stem cell-derived hNP cells and neurons could respond differently than cells of primary origins [16,17,18,19,20,41]. The observation of viral replication in hNP cells and hNP-derived neurons in this study provides further evidence that African and Asian isolates generate distinct differences in neural developmental processes.

Both isolates displayed infection in the hNP cells and neurons compared to the Vero cells. Adding equivalent volumes of ZIKV resulted in nearly 100% of the Vero cells infected with ZIKV, whereas only a fraction of hNP cells and neurons produced viral protein E (Figure 3). The relatively poor rates of infection in hNPs and neurons compared to Vero cells may be due to a functional interferon response in the CNS lines compared to the interferon deficient Vero cells [42,43]. The African-lineage virus was able to infect twice as many hNPs and mature neurons compared to Asian isolates (Figure 3). The African isolates used in this study were extensively cultured in fetal mouse brains for several decades, which may have created viral isolates more adept at infecting the primordial mammalian CNS [44]. Conversely, Asian isolates infected fewer neural cells initially, yet were able to remain in the population longer by inducing only modest cell death (Figure 1, Figure 2 and Figure 3). Perhaps this trait is an indication of the Asian ZIKV isolate’s ability to more effectively evade host anti-viral defenses [45].

The authors suspect that the African-lineage isolates produced lower viral titers because of virus-induced cell death. Only a small percentage of the hNP or neuron populations contained the ZIKV E protein, but the viability of the neural populations dramatically decreased. This result suggested that some of the cell death could be due to bystander killing rather than primary ZIKV infection (Figure 3) [46]. This hypothesis of bystander effects of ZIKV infection altering the proliferation of infected cells and the paracrine stimulation of hNP cells could trigger apoptotic pathways independent of infection [41,47]. Mutations could enable the Asian-lineage isolates to prevent cell death pathways or decrease bystander cell death. Such changes may allow Asian-lineage ZIKV to persist in the prenatal or neonatal CNS without fatal consequences [48].

Beyond evaluating ZIKV growth and viral-induced cell death, the authors tested how ZIKV infection disturbed the functional attributes of hNP cells and hNP-derived neurons. Neural progenitor cell function is critical for CNS growth and maturation [26]. Previous in vitro models reported that ZIKV caused perturbation of the cell cycle and transcription of neurodevelopmental pathways in hNP cells [17,49]. Murine experimental models and autopsies of newborns with congenital Zika syndrome exhibited altered neural cell proliferation and migration after ZIKV infection [14,15,50]. This study’s data demonstrated that SPH uniquely inhibited proliferation prior to any detrimental effect on cell viability (Figure 4A,B), suggesting the in vitro infection model could be used in further examining the mechanisms of ZIKV-induced microcephaly.

hNP cell migration is the culmination of intracellular processes and interactions with the extracellular matrix [51]. Monitoring hNP cell migration after ZIKV infection indicated that both IbH and SPH could subvert normal cell mobility prior to the cytotoxic effects of ZIKV. Deficiencies in the cell motility likely contributed to the observation of CNS malformation in mouse models when exposed to either African- or Asian-lineage viruses [11,52]. This study’s results suggested that Asian ZIKV isolates caused diminished proliferation and migration of hNP cells while avoiding significant cell death. In combination, these events likely contribute to how ZIKV infection leads to congenital brain malformations.

The architecture of the cortex is established by the proliferation and migration of hNP cells [53]. Later, neurons mature within the cortex by extending neurites and synaptogenesis [54]. The integrity of neurite structure is critical for CNS function, and the precise organization of the cytoskeleton, microtubule dynamics, and neurite outgrowth can be pathologically disrupted [55]. The impact of ZIKV infection in neurons was evaluated by measuring the degree of neurite outgrowth and branching. High MOI infections with Asian isolate SPH caused significant loss in the number of neurites, the length of the neurites, and the number of branch points. Clinical reports from congenital ZIKV infection included ocular defects and seizures [56]. The attenuated proliferation, migration, and neurite outgrowth reported in this study appears to resemble other pathologies leading to seizures and cognitive defects [53,57].

## 5. Conclusions

In conclusion, the authors’ use of well-defined cell types and assays allowed them to evaluate the functional consequences of ZIKV infection in cells of the developing brain. The direct comparison of African and Asian isolates of the ZIKV revealed the ability of the Asian ZIKV isolate, SPH, to disturb hNP and neuronal cell function. The diminished hNP cell proliferation, migration, and altered neurite outgrowth in neurons suggested that Asian-lineage ZIKV infection altered cellular function in the developing CNS while slowly inducing cytopathic effects, which could result in congenital Zika syndrome. The evidence indicates that ZIKV isolates of Asian origin may have a unique ability to alter critical cellular processes. Further work comparing viral evolutionary changes should provide evidence of how Asian isolates of the ZIKV are capable of altering cells of the developing brain without inducing extensive cell death and will help elucidate the mechanism behind ZIKV congenital syndrome.

## Supplementary Materials

The following are available online at http://www.mdpi.com/1999-4915/10/10/550/s1. Figure S1. ZIKV isolate-specific growth and cytotoxicity in hNP cells at six days post-infection. Table S1. Comparison of ZIKV isolates at unique MOI (* indicates *p* < 0.05). Table S2. Comparison of MOI within each ZIKV isolate.
