# Supplementary material for: Strain-Dependent Consequences of Zika Virus Infection and Differential Impact on Neural Development
Source: Viruses. 2018 Oct 9;10(10):550. doi: 10.3390/v10100550 (PMC6212967; doi:10.3390/v10100550)
Supplement: Supplementary file 1 [file viruses-10-00550-s001.docx]

**Supplementary Figure and Tables**


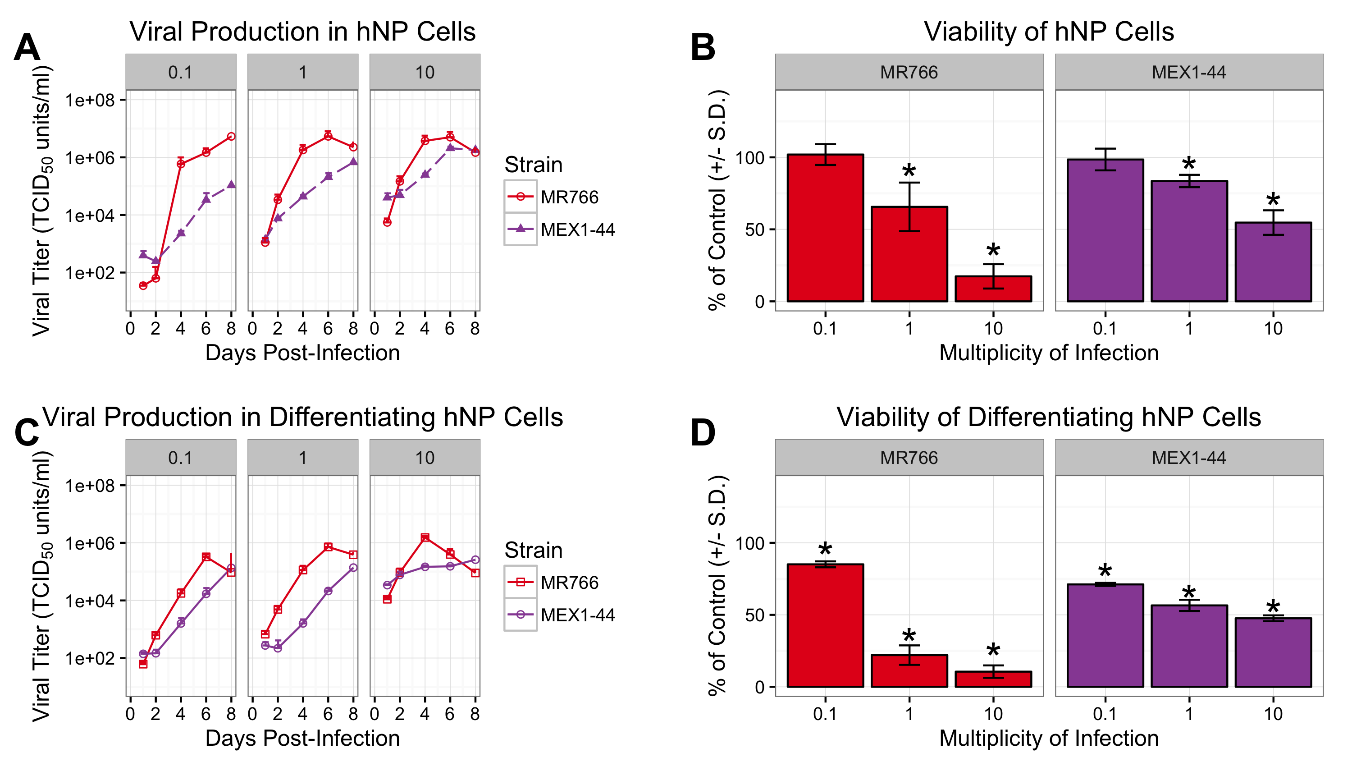


**Figure S1.** ZIKV isolate-specific growth and cytotoxicity in hNP cells at six days post-infection. (**A–D**) African-lineage ZIKV isolate (MR766) grew robustly and induced cell death in undifferentiated hNP and differentiating hNP cells, whereas Asian isolate (MEX1-44) replicated more slowly with less extensive cell death. * demonstrates *p* < 0.05.

**Table S1.** Comparison of ZIKV isolates at unique MOI (* indicates *p* < 0.05)

| **MOI** |  | | | | |
| --- | --- | --- | --- | --- | --- |
| 0.1 |  | **Mex-144** | **MR766** | **IbH** | **SPH** |
|  | **Mex-144** |  |  | * |  |
|  | **MR766** |  |  | * |  |
|  | **IbH** | * | * |  | * |
|  | **SPH** |  |  | * |  |
| 1 |  | **Mex-144** | **MR766** | **IbH** | **SPH** |
|  | **Mex-144** |  |  | * |  |
|  | **MR766** |  |  |  |  |
|  | **IbH** | * |  |  | * |
|  | **SPH** |  |  | * |  |
| 10 |  | **Mex-144** | **MR766** | **IbH** | **SPH** |
|  | **Mex-144** |  | * | * |  |
|  | **MR766** | * |  |  |  |
|  | **IbH** | * |  |  | * |
|  | **SPH** |  |  | * |  |

**Table S2.** Comparison of MOI within each ZIKV isolate

| Strain |  | | | | |
| --- | --- | --- | --- | --- | --- |
| SPH |  | **0** | **0.1** | **1** | **10** |
|  | **0** |  |  | * | * |
|  | **0.1** |  |  |  | * |
|  | **1** | * |  |  |  |
|  | **10** | * | * |  |  |
| Mex-144 |  | **0** | **0.1** | **1** | **10** |
|  | **0** |  |  | * | * |
|  | **0.1** |  |  |  |  |
|  | **1** | * |  |  |  |
|  | **10** | * |  |  |  |
| IbH |  | **0** | **0.1** | **1** | **10** |
|  | **0** |  | * | * | * |
|  | **0.1** | * |  |  |  |
|  | **1** | * |  |  |  |
|  | **10** | * |  |  |  |
| MR766 |  | **0** | **0.1** | **1** | **10** |
|  | **0** |  |  | * | * |
|  | **0.1** |  |  |  | * |
|  | **1** | * |  |  | * |
|  | **10** | * | * | * |  |
